# Methods for the metrological characterization of wearable devices for the measurement of physiological signals: state of the art and future challenges

**DOI:** 10.1016/j.mex.2023.102038

**Published:** 2023-01-23

**Authors:** G. Cosoli, L. Antognoli, L. Scalise

**Affiliations:** Department of Industrial Engineering and Mathematical Sciences, Università Politecnica delle Marche, v. Brecce Bianche, Ancona 60131, Italy

**Keywords:** Wearable devices, Metrological characterization, Test protocol, Measurement accuracy, Validation, Test protocol for the validation and the metrological characterization of wearable sensors for physiological monitoring

## Abstract

Wearable devices are rapidly spreading in many different application fields and with diverse measurement accuracy targets. However, data on their metrological characterization are very often missing or obtained with non-standardized methods, hence resulting in barely comparable results. The aim of this review paper is to discuss the existing methods for the metrological characterization of wearable sensors exploited for the measurement of physiological signals, highlighting the room for research still available in this field. Furthermore, as a case study, the authors report a customized method they have tuned for the validation of wireless electrocardiographic monitors. The literature provides a plethora of test/validation procedures, but there is no shared consensus on test parameters (e.g. test population size, test protocol, output parameters of validation procedure, etc.); on the other hand, manufacturers rarely provide measurement accuracy values and, even when they do, the test protocol and data processing pipelines are generally not disclosed. Given the increasing interest and demand of wearable sensors also for medical and diagnostic purposes, the metrological performance of such devices should be always considered, to be able to adequately interpret the results and always deliver them associated with the related measurement accuracy.

• The sensor metrological performance should be always properly considered.
• There are no standard methods for wearable sensors metrological characterization.
• It is important to define rigorous test protocols, easily tunable for specific target applications.

## Graphical abstract

**Figure:**
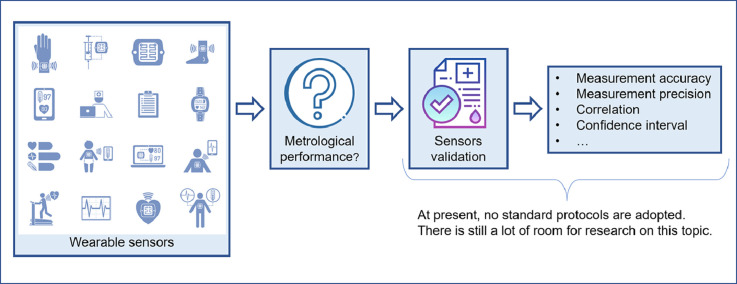

Specifications table

**Table:** 

Subject area:	Engineering
More specific subject area:	Measurement of physiological signals with wearable sensors
Name of your method:	Test protocol for the validation and the metrological characterization of wearable sensors for physiological monitoring
Name and reference of original method:	N.A.
Resource availability:	N.A.

## Overview

**Fig. 1:**
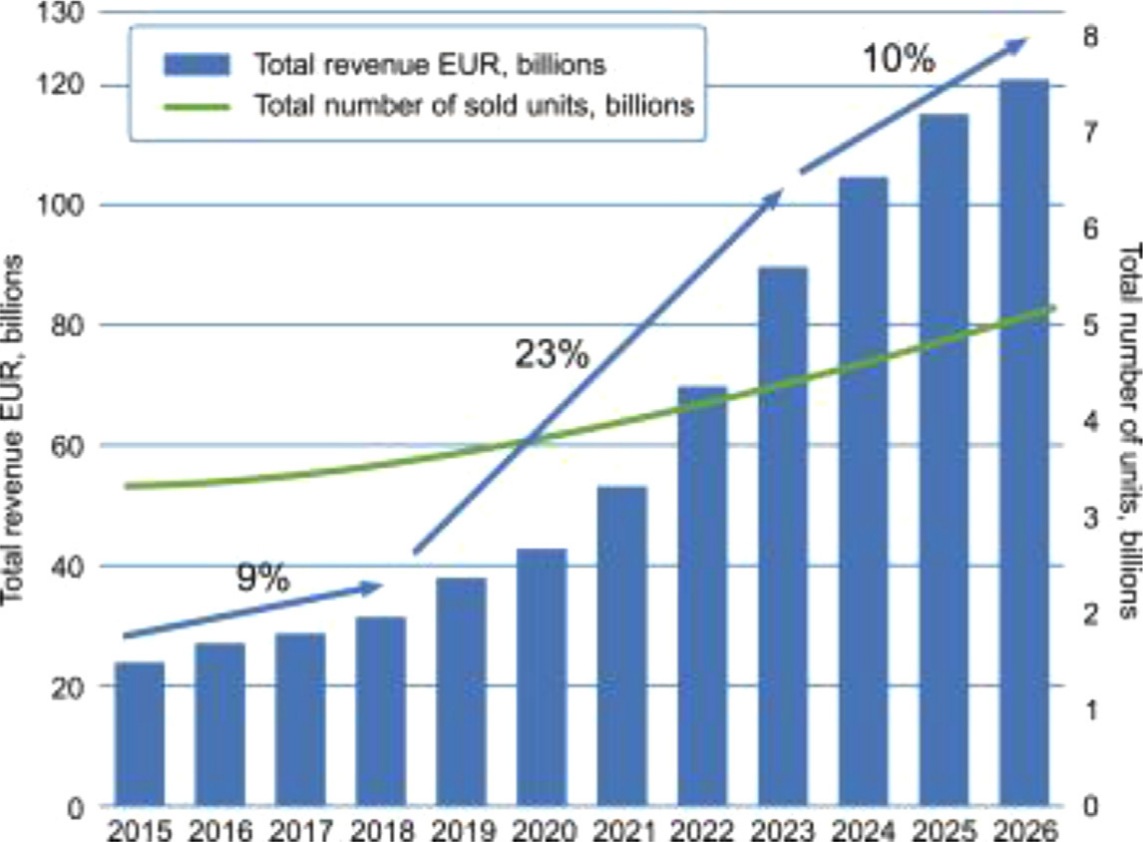
Wearable sensors growth between 2015 and 2026: forecasts in terms of revenue (blue trend line) and sold units (green trend line) [4].

Wearable sensors are spreading worldwide, with a growth frequency >20% per year, expecting to increase to 150 billion EUR by 2028 [1], for a total of 3,8 billion $ in 2022 [2]. Their growth in terms of Compound Annual Growth Rate (CAGR) has been estimated at 11.8% in the period between 2019 and 2026, with an increase of units up to over 5 billion in 2026 (Fig. 1) [3].

Indeed, they have multiple advantages: they are user-friendly, relatively inexpensive, neither invasive nor intrusive, and available in several market segments fulfilling diverse users’ needs. This is particularly true for wrist-worn devices (i.e. smartwatch-like), which at present are very common among citizens (e.g. in America 21% of citizens between 18 and 49 years wear a smartwatch or a smartband [5]). There is a plethora of application fields: from health [6,7] to industry [8,9], through sport [10,11], and rehabilitation [12,13], just to cite some. The role of wearable sensors in the remote monitoring of physiological parameters is pivotal, given their capability to acquire data 24 hours a day, 7 days per week; for this reason, their use has been often combined to Artificial Intelligence (AI) techniques for both classification and regression purposes [14,15]. This represents a huge potential to support clinical decision-making processes and health personnel in adapting therapeutic strategies and optimizing patients’ management, taking care of their actual physiological state as monitored not only in outpatient visits, but also at home and, more generally, in everyday life, hence in a continuous manner. This has become clear during the COVID-19 pandemic [16], when the potentiality of IoT-based solutions for data processing and analysis in determining health conditions has been underlined [17].

Many physiological parameters can be measured (directly or indirectly, through estimation processes that sometimes are guided by AI algorithms), such as heart rate (HR) and its variability (HRV) [18], [19], [20], [21], energy expenditure [22,23], blood pressure [24,25], breathing activity [26,27], thermal comfort [28,29], etc. Such parameters are very relevant since they can depict the overall health status of a subject and also support the early detection of pathological symptoms, hence limiting also the risk of contagion [30]. Most of the wrist-worn sensors are based on photoplethysmography (PPG), hence there is an optical signal assessing the blood volume pulsing in the underlying tissues. Consequently, several interfering factors should be thoroughly taken into account, such as the intensity and wavelength of light [31,32], contact pressure [33], sensor-skin interface, movement artefacts [34], skin tone [35], etc. All these uncertainty sources obviously impact on the measurement results, determining the uncertainty value and, hence, the sensor accuracy. On the other hand, flexible wearable sensors are gaining particular attention, as they are comfortable for long-term monitoring and can be used for a plethora of different applications, resulting promising also for medical fields [6]. Measurement accuracy determination is fundamental also for this type of wearable sensors. Thus, the Authors decided to focus this paper on the validation procedures to be applied to wrist-worn wearable sensors for the measurement of physiological parameters, with a particular attention to the assessment of cardiovascular parameters (e.g. extracted from an electrocardiographic, ECG, or photoplethysmographic, PPG signal), being them pivotal in the evaluation of both the subject's health status and well-being [36]. An example on this application has been reported as an Authors’ case study before concluding the paper.

## How to determine the measurement uncertainty of wearable devices?

The determination of the measurement uncertainty of wearable sensors is a complex process that requires thorough attention; as above-mentioned, the focus of the present paper is on wearable sensors for the measurement of cardiovascular related signals (e.g. ECG, HR, HRV, blood oxygen saturation, etc.), but also literature studies investigating diverse types of physiological signals (e.g. accelerations and respiratory rate) were considered to underline the methods adopted for the sensors characterization from a metrological point of view.

The procedure to follow for measurement accuracy and precision determination is not straightforward, since many factors need to be considered and their effect can also be synergistic; standard procedures like Monte Carlo simulation method can be exploited to analyze the uncertainty propagation. It is clear that the performance requirements vary with the target applications, especially for what concerns the metrological performance of these devices. For example, medical applications undoubtedly need higher measurement accuracy and precision than fitness tracking or personal monitoring purposes [37].

Validation procedures for wearable sensors are fundamental; however, there are no widely accepted standards to follow and literature on this topic is quite inhomogeneous; information on the wearables metrological performance is rarely available and, even when obtainable, the evaluation method is usually not disclosed, making impossible to replicate the test. In fact, each study is performed with a personalised test protocol, tuned for the target application and characterization objectives; nevertheless, many different aspects vary among protocols, such as test population characteristics, test protocol, measured quantities, performance metrics (e.g. mean absolute percentage error, correlation coefficient, confidence intervals, etc.) and so on. This creates barely comparable results [38]; on the contrary, it would be extremely important to define a standard to adopt, in order to start building a database with results for all the tested devices, even from different laboratories. Indeed, some associations have started to define recommendations and guidelines in this fields, such as the Consumer Technology Association (CTA) and the American National Standards Institute (ANSI), who in 2018 wrote in conjunction the ANSI/CTA Standard “Physical Activity Monitoring for Heart Rate” (ANSI/CTA-2065) [39]; this document provides indications for test population (in terms of skin tone, BMI, gender, age, etc.), testing environment, reference devices, and test protocol. However, literature studies have not implemented these recommendations in a systematic way, so at present the materials and methods followed in validation studies are very diverse between each other.

Some of the present authors have spent many efforts in recent years to analyze the state of the art on wearable sensors characterization, trying to define test protocols and evaluation procedures that can be relatively easily scaled for different applications, maintaining a rigorous method in metrological evaluation. In particular, in 2020 Cosoli et al. [38] published a review paper on the accuracy and metrological characteristics of wrist-worn and chest-strap devices; first of all, from literature it is clear that there is a plethora of physiological variables being measured through wearable sensors, even if most of the studies focus on step counting, energy expenditure, HR, and sleep. Given their ease of use and the similarity with a common watch, wrist-worn devices are prevailing on chest-strap ones, which conversely are usually more accurate (especially in high-intensity activities [40]). In any case, it is evident that almost each study develops its own test protocol to verify the hypothesis of interest, also reporting the results in different ways; what is more, sometimes the test device is compared to a reference device (e.g. a medical grade system), whereas in other cases another wearable device, reported to be “more accurate”, is employed. This inevitably leads to inhomogeneous results. Also the characteristics of the test population are very different among studies, in terms of numerosity, age, health conditions, BMI, and so on; this impact also on the suitability of the test device for the specific test sample, since many sensors are available in different sizes that should fit the subject wearing them. Regarding the assessment of accuracy, there are no common evaluation metrics reported in literature; among the most used, we can cite the absolute percentage error (APE) – or the mean APE (MAPE) – and the correlation coefficient. However, it would be important also to measure the statistical confidence of the measurement results, for example considering the confidence interval (e.g. at 95%, i.e. CI95%) or the distribution of measurement differences (residuals), describing how much precise (values are close to each other) and accurate (measures are near the expected value) the measurement is.

As an explicative example, the authors report a case study in the following, considering a wearable sensor for the measurement of ECG signal.

### The important role of physiological variability

Particular attention should be paid to physiological variability, which cannot be avoided and undoubtedly impacts on both measurement results and evaluation procedures; indeed, whereas in mechanical or electrical measurements repeated measures can be performed on the same measurand in the same conditions, when evaluations are carried out on a human being the subjectivity plays a pivotal role that cannot be ignored [41]. Hence, given the physiological variability intrinsic of human measurands, it would be important to change the point of view: again, we are not in front of mechanical, electrical, or thermal quantities, whose measurement can be repeated. Consequently, traditional procedures for evaluating measurement uncertainty, like those described in the Guide to the expression of Uncertainty in Measurement (GUM [42]), cannot be applied as-are for the metrological characterization of sensors measuring physiological signals on human beings. An alternative can be the exploitation of different techniques, mainly standard statistical techniques, allowing to estimate the measurement accuracy and precision, along with agreement with a reference device (gold standard). It is worthy to underline that the obtained metrics will depend on the test protocol characteristics (e.g. sensor positioning or physical activity intensity), but also on the sample parameters (especially when PPG sensors are involved, being them sensitive to body composition and skin tone [35] – but also BMI influences the accuracy computation [43]).

### How to validate a new prototype from a metrological point of view?

What if a research group or a producer decides to develop a new prototype for the measurement of physiological parameters? First of all, since the very early stage of the design, it is pivotal to take into account recommendations, guidelines, and standards valid in the Countries of interest for the marketing of the new product. Considering the European Union, a device has to be compliant with the applicable directives in order to obtain the European Conformity marking (CE), for example regarding electromagnetic compatibility. This regards the safety of the device, but another fundamental aspect concerns accuracy, when the instrument is intended as a sensor. In this case, the evaluation can be done in two main different ways: (1) considering a patient simulator (which, however, is not practical in case of PPG-based sensors) or (2) comparing the measurement results with a gold standard. For wearable sensors, the latter is the most pursued way. In the next section, some examples from literature are reported.

### Some studies on validation from literature: how tests are performed?

As above-mentioned, in literature the test procedures are quite different; for an overall view until 2020, the authors recommend to refer to the previous review paper published in Measurement journal [38]. On the other hand, in this section some examples from 2020 onwards are reported in order to provide the reader with a brief overview of the current state of the art, focusing more on the adopted test validation procedures than on the sensors performance results.

The importance of validating wearables to provide accurate results are highlighted by Jourdan et al. [44], focusing on the role of Machine Learning (ML) techniques in the validation of sensors to monitor gait. They found that in more than half of the considered studies the ground truth is represented by annotations and not by a reference instrument; they also stressed the lack of standard evaluation metrics as well as the inhomogeneity in terms of acquisition context. They stressed the need of defining validation protocols (including sensors, test environment, test periods, test population size, etc.), stating that it is pivotal to compare wearable results with those obtained with a reference instrument. This way, exploiting ML algorithms it is possible to make reliable predictions for diagnosis purposes. The importance of validating the sensors against a gold standard (both in laboratory and in real-life conditions) has been evidenced also by Kuo et al. [45] in the fields of sport and military applications. Some literature studies and the related results are reported hereafter, summarizing their findings in Table 1.

**Table 1:** Results from the considered literature studies.

Refs.	Wearable sensor	Positioning	Measurand	Test population and procedure	Results
Na and Buchanan [46]	3D Inertial Measurement Unit (IMU)	Femur, tibia, pelvis, iliac spine, S2 sacral ridge	Acceleration	39 participants walking at controlled speed gaits	Spearman's rho: −0.63 (*p* < 0.01)
Kant et al. [47]	Patch sensor	Chest	HR, respiratory rate (RR)	94 obese patients during and after bariatric surgery	Mean difference (standard deviation): 1.26 (0.84) bpm for HR, 1.78 (1.90) bpm for RR
García Patiño and Menon [48]	Inductive strain textile sensor	Back	Strain	Theoretical calculations and simulations using a healthy subject's anthropometric dimensions	- (comparison between simulated and theoretical inductance)
Singh et al. [49]	Patch wireless ECG prototype	Chest	HR, RR intervals	ECG simulator at 30, 60, 120, and 240 bpm	Accuracy: 100% at 30 and 60 bpm, 99.8% at 120 bpm, and 98.8% at 240 bpm for HR; 100% at 30 and 60 bpm, 98.0% at 120 bpm, and 96.05% at 240 bpm for RR
Vescio et al. [50]	Wrist-wearable watch connected to wired muscle sensors	Arm	Resting tremor pattern	21 subjects with alternating/synchronous resting tremor pattern	Good level of agreement; *r* = 0.93 for tremor frequency and *r* = 0.92 for phase difference (*p* < 0.001)
García-Villamil et al. [51]	6-axis IMU	Foot	Walking speed	21 participants walking on different grounds	ICC = 0.69 (*p* < 0.001)

The analysis of gait and, in particular, acceleration data associated with instability, is the focus of the study conducted by Na and Buchanan [46], underlining the importance of objective data (gathered through wearable sensors) with respect to self-assessment by patients; however, no gold standard instruments were considered and only the relationship with self-perceived instability was evaluated for validation scopes.

Validation studies of wearable sensors have been made also in clinical settings; for example, Kant et al. [47] evaluated the agreement between a wearable patch sensor and a reference monitor in the measurement of both HR and respiratory rate. The test population consisted in 94 obese patients; they define the agreement as “the mean absolute difference between monitoring devices” and evaluated it through Bland-Altman plot and Clarke Error grid analysis. They also estimated the reliability of the patch sensor computing the amount, the duration, and the causes of data loss. In this case, the test protocol is well defined and foresees the exploitation of standard validation techniques; however, the test population is quite peculiar and results could be not easily generalizable to the general population.

Sensor validation is obviously fundamental also when a new sensor is developed, starting from its design and proceeding with the prototype realization. For example, García Patiño and Menon [48] developed an inductive textile wearable sensor and described the validation procedure; however, their validation consists in a comparison between initial theoretical calculations and simulation data, whereas no tests on a real population were performed. Singh et al. [49] designed and validated a smartphone-based wearable monitoring system for cardiac activity; the performance in measuring ECG signal is quantified by calculating the percentage error in RR intervals, using a patient simulator as reference. Moreover, they also conducted a clinical study to compare the developed prototype and a gold standard instrument. Similarly, Vescio et al. [50] developed and validated a wearable sensor for detecting resting tremor in Parkinsonian individuals or essential tremor; the results were compared with a standard electromyograph and agreement was evaluated. The test population consisted in 14 patients with Parkinson's disease; Bland-Altman plot and correlation were used for the analysis. Again, the target application is very specific, but the protocol is well-described in terms of both gold standard device and data analysis and performance metrics. García-Villamil et al. [51] validated a 6-axis wearable inertial sensor for gait assessment, comparing the measurement results with a reference sensor; they evaluated the accuracy through the computation of intraclass correlation coefficient. Therefore, the adopted method can be considered meticulous and rigorous.

To conclude this section, it is worthy to note that also the definition of the validation procedure itself is not unique, since experimental tests are not always performed and the evaluation methodologies are not clear, not even standardised. For sure, each target application requires a fine-tuned validation protocol, but some characteristics should be standardised, such as ground truth type, test conditions, test population requirements, evaluation metrics, etc. The main limitations evidenced in literature are the absence of guidelines and standardised recommendations, aiming at creating homogeneous results in terms of metrological characterization of wearable devices. It is important to develop procedures specific for wearable sensors, which are inevitably different from other sensor types, consequently requiring specific test protocols. Moreover, data processing techniques should be well defined, also foreseeing the exploitation of ML or AI technologies, which are nowadays commonly employed in a plethora of applications.

### Data management: how to guarantee data privacy and security?

Another important aspect to be thoroughly taken into account is data management in accordance with the General Data Protection Regulation (Regulation 2016/279, commonly known as GDPR), in order to guarantee the privacy and the security of the gathered data. In this sense, all the participants to a validation study should sign an informed consent module after that test aims and modalities have been clearly explained and eventual doubts have been solved. The Ethical Committee should be involved after starting an experimental campaign when necessary; however, all the test should be always performed in compliance to the WMA Declaration of Helsinki [52].

## Between standardization and customization needs

If the need for standardization in validation procedures is clear in order to obtain comparable results (at least the way in which data are processed and uncertainty related outputs are provided), from a different point of view the test protocols should be adapted to the specific target applications (e.g. distinction between sport and medical devices, surely requiring different metrological performances). Indeed, different target applications require customised test protocols; just as an example, let us consider the case of monitoring of swimming athletes [53]: diverse test conditions are present, different physical activities should be envisaged. Hence, the developed test protocols should be “scalable” and flexible according to the particular application for which the wearable sensor is intended.

In any case, it is fundamental to always thoroughly report the followed evaluation procedures, including data acquisition modalities and data processing techniques, together with the test conditions and the characteristics of the test population, so as to make the whole validation procedure replicable.

Even if test protocols should be tuned for peculiar applications, some cornerstones should be considered, for examples those reported in Table 2. Measurement accuracy, precision, and statistical confidence of a wearable sensors should be always provided, to give the end-user important indications on the results reliability, as well as to indicate researchers where there is still room for improvement.

**Table 2:** Requirements for validation protocol.

Item	Description
Test population	The test population should be described in terms of gender, age, weight and height (or BMI), skin tone, etc.
Test protocol	The test protocol should be reported in all its phases, with sufficient details to be easily replicable.
Ethics approval	Eventual ethics approval number should be reported. Anyhow, something on how the tests were performed to avoid ethical issues should be stated. Also, informed consent should be mentioned.
Data processing and output variables	All data processing tools, software, and methodologies should be thoroughly reported. Also output variables of interest should be underlined, trying to exploit standard evaluation methods/parameters.
Performance metrics	A wearable device should be characterized at least in terms of measurement accuracy, precision, and statistical confidence. Additional metrics should be described in detail and reported as well.

### The authors’ case study

As a case study, the authors would like to finally report a test protocol for the metrological characterization and, hence, validation of wireless wearable sensors; in particular, a sensor for the electrocardiographic (ECG) monitoring has been considered, given the importance of the information that can be inferred from this signal.

### Aim

The objective of the reported study is the metrological characterization of Samsung Galaxy Watch3 (which can record ECG signal through 3 electrodes, providing a 30-s acquisition) with respect to BioHarness 3.0 (reference device, with an accuracy of ± 1 bpm for HR in the measurement range of 0–140 bpm; sampling frequency: 250 Hz).

### Test protocol

**Fig. 2:**
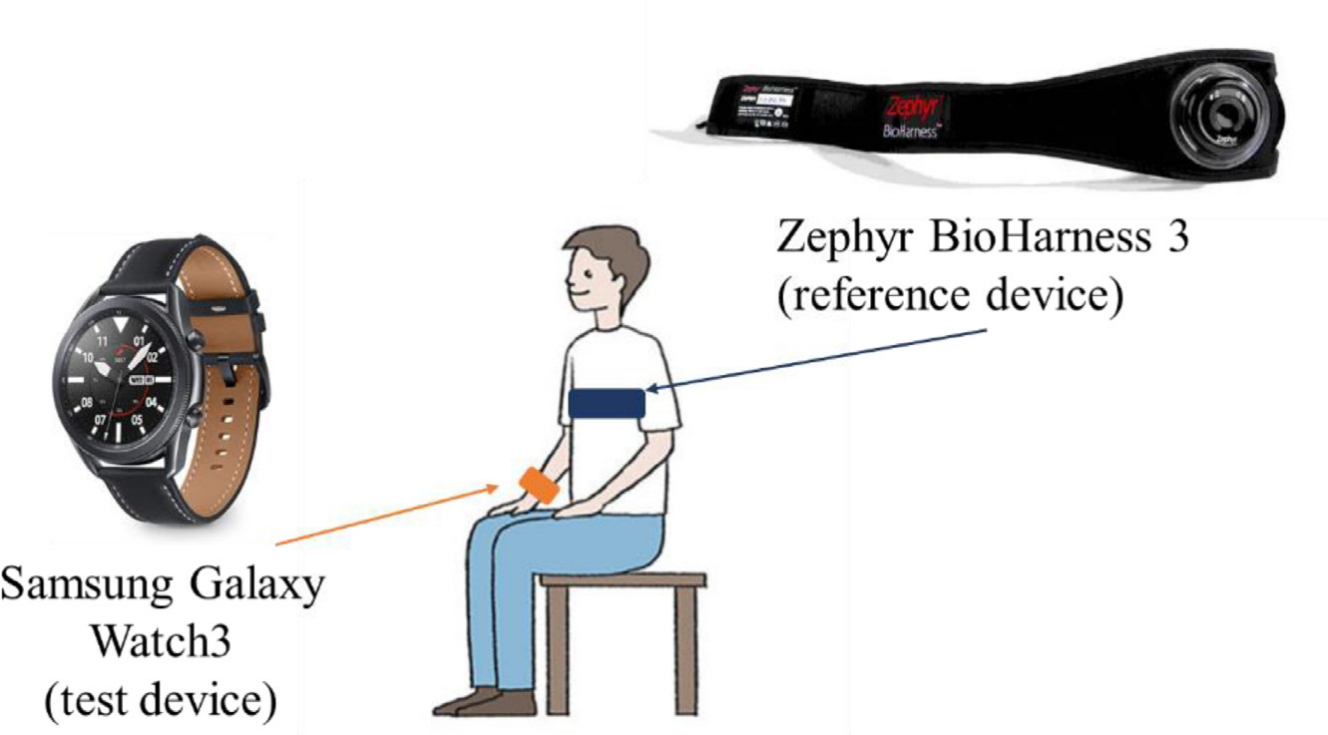
Test setup, with test (Samsung Galaxy Watch3) and reference (Zephyr BioHarness 3.0) devices worn by the subject.

**Fig. 3:**
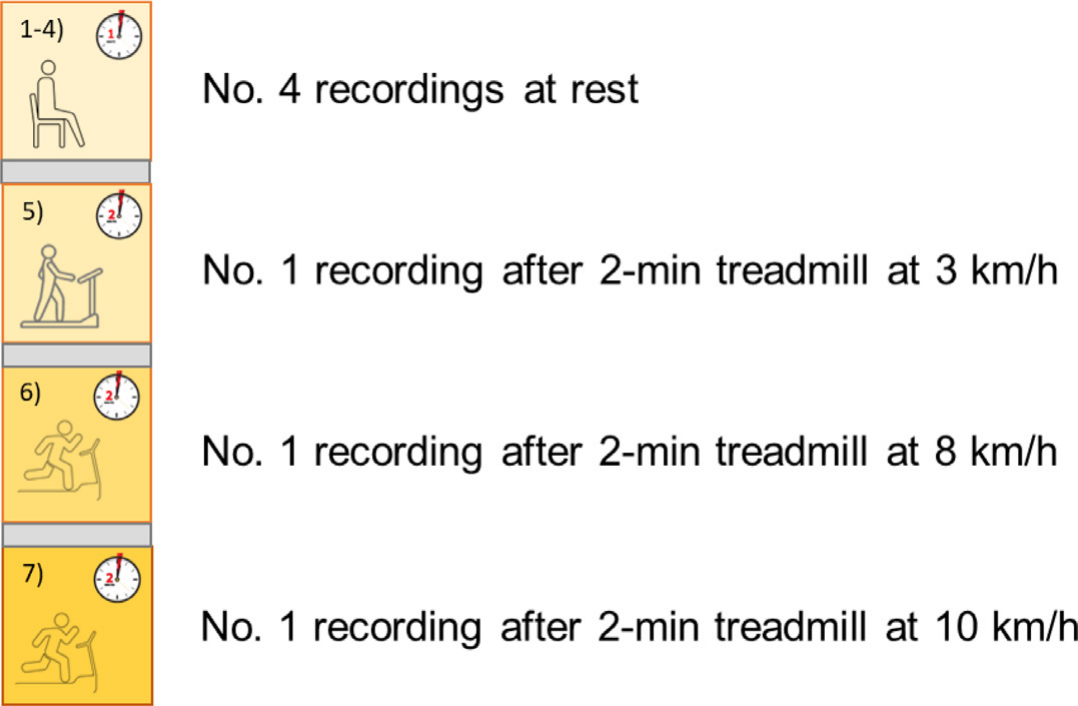
Test protocol: acquisition phases.

Each subject simultaneously worn the two devices, as reported in the experimental setup (Fig. 2).The test device was evaluated with respect to a reference device; the protocol involved ECG acquisitions in 2 different conditions: 1) at rest (4 recordings) and 2) after physical exertion (2-min treadmill at 3–8–10 km/h, with 0-slope – 3 recordings), as reported in Fig. 3. In this way, it is possible to enhance the variability of the measured signal and, hence, evaluate the metrological performance in a wider range. It is worthy to underline that all the acquisitions were made in static conditions, on the subject staying as still as possible to minimize movement artefacts.

### Data processing

Concerning data processing, the Pan-Tompkins’ algorithm was employed for R peaks detection and tachogram was derived from test and reference signals. Measurement deviations (i.e. differences between HR measured by test and reference devices) were computed and analyzed according to standard statistical methods: (1) distribution of deviations (computing mean, µ, and standard deviation, σ, values), (2) analysis of agreement [54], and 3) correlation between test and reference signals.

### Test population

The test population consisted of 10 healthy volunteers, aged (21±2) years (mean ± standard deviation), with a Body Mass Index (BMI) of (21.53±2.21) kg/m^2^; a total of 7 recordings was performed on each subject, for a total of 70 recordings. All the subjects were made sign an informed consent module after that the aims and methods of the study were clearly described. Moreover, all the tests were performed in accordance with the WMA Declaration of Helsinki [52].

## Results

**Fig. 4:**
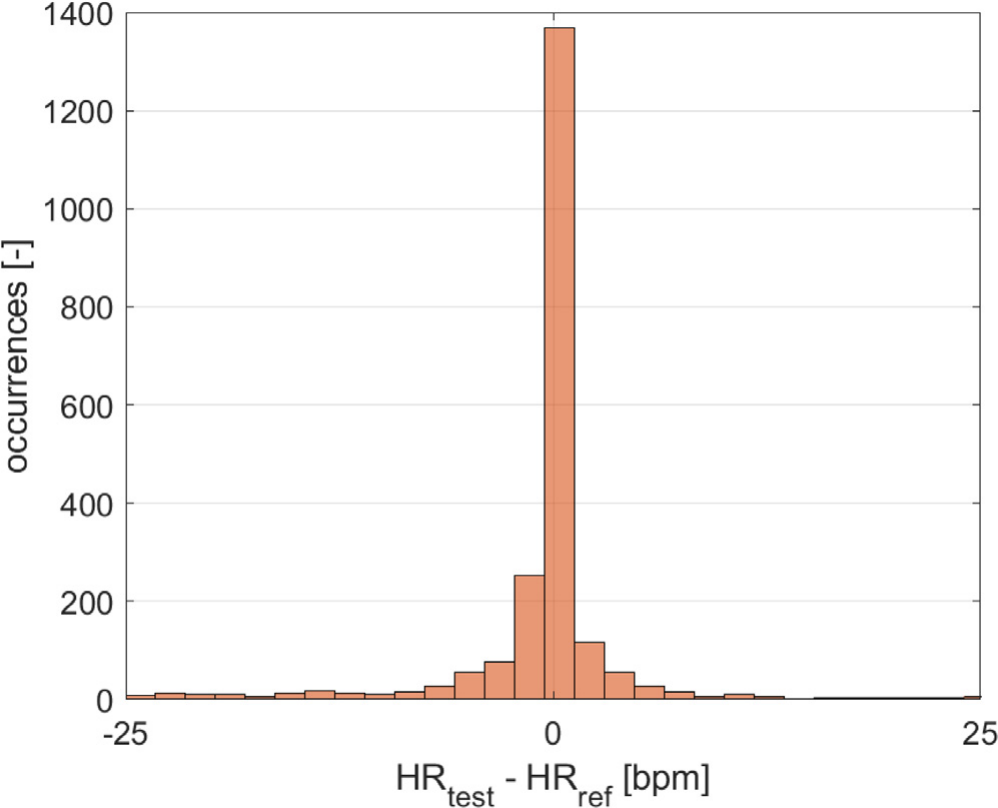
Distribution of measurement deviations (study case).

**Fig. 5:**
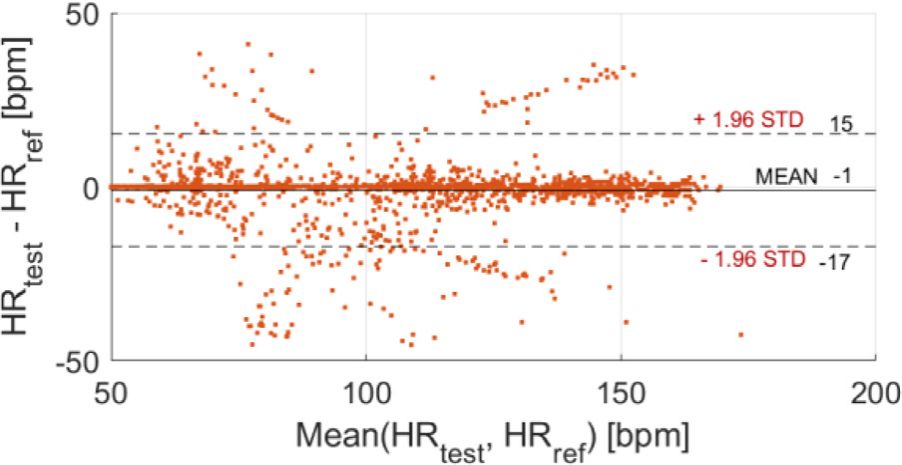
Data agreement, Bland-Altman plot (case study).

**Fig. 6:**
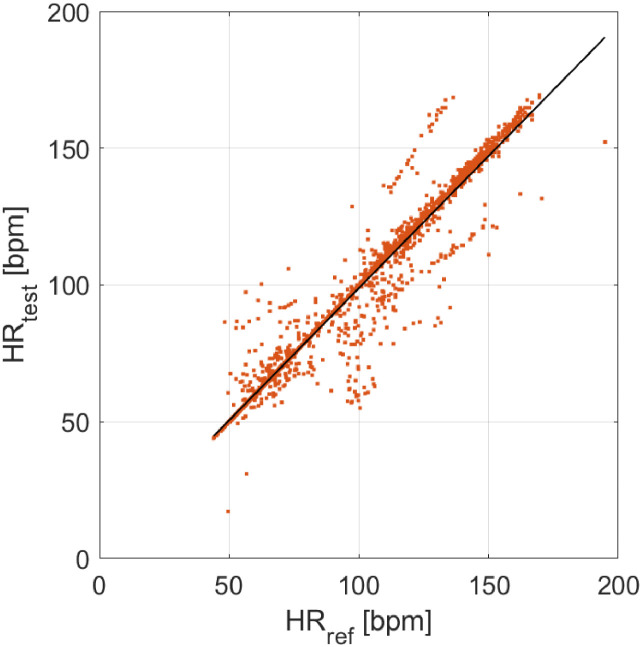
Correlation between test (y-axis) and reference (x-axis) data.

The results in terms of distribution of measurement deviations, data agreement (Bland-Altman plot), and correlation are reported in Fig. 4, Fig. 5, and Fig. 6, respectively. The test device appears to be very accurate and quite precise in the HR assessment, providing a mean difference of −1 bpm, with a standard deviation of 8 bpm. The analysis of data provided an average value of measurement deviation (bias) equal to −1 bpm, which can be related to measurement accuracy. In relation to the measurement precision, a confidence interval at 95% of [−17, 15] bpm (mean ± standard deviation, with a coverage factor *k* = 2) was obtained; a slightly lower precision can be observed for higher HR values. However, the test device is in agreement with the reference one. The Pearson's correlation coefficient is equal to 0.97, demonstrating a strong linear correlation with the reference sensor.

### Discussion and conclusions

The proposed test protocol proved to be easy to realize and suitable to investigate the metrological performance of a test device in a quite wide measurement range (obtained through acquisitions performed both at rest and after physical activity). Measurement accuracy, precision, and statistical confidence of HR measurement were obtained, providing a complete picture of the device metrological performance. This type of investigation is fundamental to validate and characterize from a metrological point of view new sensors for the cardiac activity monitoring, hence providing results together with information on measurement accuracy. In future, the defined test protocol will be applied to other ECG monitors, both commercially available and new developed prototypes. Besides R peaks and related tachogram, also other features from ECG waveform (e.g. amplitude, P, Q, S, and T peaks locations, frequency content, etc.) will be evaluated, together with coherence with the ECG waveform provided by reference device. Also, particular attention should be paid to the proprietary algorithms of the investigated devices, since artificial corrections made on data could introduce artefacts in the final results (e,g. the aligned points visible in Fig. 6).

The procedures described in this case study can be generalized and adapted to different wearable sensors measuring other physiological signals (e.g. for gait analysis); however, the basic scheme can be considered still valid, as well as the data processing methodologies and the presentation of the obtained results.

## Conclusions

In conclusion, this paper aims at underlining the importance of thoroughly report the test protocol and the data processing methods used to validate a wearable sensor exploited for the measurement of physiological signals. Literature is quite inhomogeneous, however the metrological characterization of wearable devices is fundamental to properly interpret the provided results and rigorous attention should be paid to these aspects. Both researchers and manufacturers should become confident with these topics and provide information as clear as possible, adopting output variables of common use and properly considering all the factors possibly interfering with their results. In the monitoring of physiological parameters, the human factor and the related variability cannot be avoided; hence, the perspective should be changed and suitable methods should be adopted to deal with physiological variability, still obtaining reliable results.

## Ethics statements

All the results reported in the “The authors’ case study” section were anonymised. The Research Ethics Committee of Università Politecnica delle Marche certified that the research study was compliant with the university Research Integrity Code. Moreover, tests were carried out according to the WMA Declaration of Helsinki and all the subjects signed an informed consent module.

## CRediT authorship contribution statement

**G. Cosoli:** Conceptualization, Methodology, Investigation, Visualization, Writing – original draft, Writing – review & editing. **L. Antognoli:** Methodology, Investigation, Writing – review & editing. **L. Scalise:** Conceptualization, Methodology, Investigation, Visualization, Writing – review & editing, Supervision.

## Declaration of Competing Interest

The authors declare that they have no known competing financial interests or personal relationships that could have appeared to influence the work reported in this paper.

## Data Availability

Data will be made available on request.
